# Inhibiting hedgehog signal by a patched-1 antibody

**DOI:** 10.1038/s41421-025-00772-6

**Published:** 2025-03-25

**Authors:** Qinli Hu, Xiaofeng Qi, Linda Donnelly, Xiaochun Li

**Affiliations:** 1 Department of Molecular Genetics, University of Texas Southwestern Medical Center, Dallas, TX USA; 2 Department of Molecular Biology, University of Texas Southwestern Medical Center, Dallas, TX USA; 3 Department of Biophysics, University of Texas Southwestern Medical Center, Dallas, TX USA

**Keywords:** Cryoelectron microscopy, Extracellular signalling molecules

Dear Editor,

The Hedgehog (HH) signaling pathway is crucial for the developmental patterning of animal embryogenesis and the regeneration of adult tissues^1^. The aberrant activation of HH signaling has been linked to various cancers, such as basal cell carcinoma and medulloblastoma^1^. Consequently, investigations into HH signaling not only contribute to understanding the molecular mechanisms underlying animal development but also offer valuable insights into potential cancer treatment approaches. Particularly, Smoothened (SMO, Class-F GPCR as an HH signal transducer) inhibitors, such as Vismodegib and Glasdegib, have been used for the treatment of basal cell carcinoma and acute myeloid leukemia in clinics^2^.

The Patched-1 (PTCH1), a negative regulator of HH signaling, contains 12 transmembrane helices (TMs) and two extracellular domains (ECDs)^3–7^. Several studies showed that PTCH1 functions as a cholesterol transporter to maintain the low concentration of cholesterol in cilia^4,8^. Our previous studies showed that one Sonic Hedgehog protein (SHH) concomitantly employs its two different epitopes and binds two PTCH1 receptors in an asymmetric manner^9^ and this complex eventually triggers the endocytosis of PTCH1^10^ to elevate cholesterol concentration in cilia^8^. SMO, a downstream class-F GPCR, then traffics to the cilia and uses its intramolecular channel to bind the free cholesterol on the cilia membrane for signaling activation^11,12^.

HH, PTCH1 and SMO are potential targets to modulate HH signaling. A specific HH antibody named 5E1 can block the HH signal via interfering with HH–PTCH1 interaction^13^. Remarkably, a nanobody (Tl23), which binds to the ECD1 of PTCH1, has been shown to induce conformational changes of the ECDs and disrupt the cholesterol transport channel of PTCH1, thus acting as an agonist of HH signaling^14^. It remains unclear whether a reagent that targets the ECDs of PTCH1 to prevent HH binding without triggering the conformational change of its cholesterol transport channel can act as an antagonist of HH signaling. This reagent may inhibit the HH signal, helping to suppress excessive HH signaling and potentially help treat HH-related human diseases.

After immunizing mice with an engineered human PTCH1 protein (termed PTCH1*, a functional PTCH1 variant with two cytosolic loops truncated^6^), several anti-PTCH1 monoclonal antibodies (mAbs) had been validated to recognize the native PTCH1 protein by pull-down assay (Fig. 1a), but not bind to denatured protein by western blotting (Supplementary Fig. S1). To explore the binding epitopes of these antibodies on PTCH1, we performed pull-down assays to test the interaction between PTCH1 and each mAb in the presence of His-tagged SHH ligand (SHH-N). The results showed that three of the mAbs, 4G8, 4H2, and 6H3, can compete with SHH-N for PTCH1 binding (Fig. 1a). However, these Abs could not inhibit the HH signal in SHH-Light II cells^6^; in contrast, all three Fabs at a final concentration of 60 μg/mL are capable of suppressing the HH signal compared to the control anti-Niemann–Pick C1 protein Fab^15^ (Fig. 1b; Supplementary Fig. S2). Therefore, we purified the Fabs for further signaling and structural investigations.

**Fig. 1:**
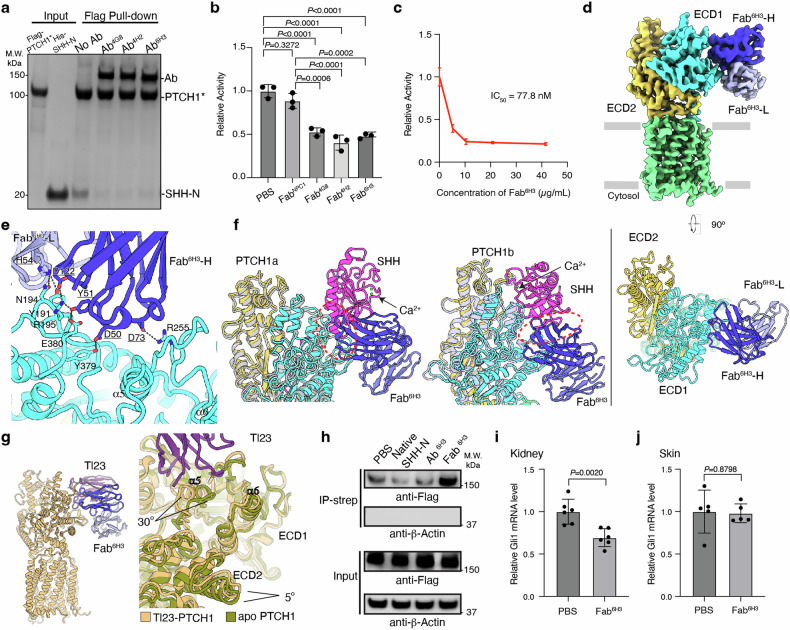
Cryo-EM structure of Fab^6H3^ bound to human PTCH1 and its inhibition of HH signaling. **a** Pull-down competition assay with monoclonal antibodies (mAbs) competing with SHH to bind PTCH1. The proteins are detected by Coomassie staining. **b** HH signaling assay in the presence of Fabs. The HH signal was measured using Dual-Luciferase Reporter Assay in SHH-Light II cells. PBS buffer and an anti-NPC1 Fab (Fab^NPC1^) are used as negative controls. Data are represented as mean ± SD (*n* = 3). One-way ANOVA was performed between the control groups and each of the experimental group by GraphPad Prism 10. *P* values are labeled. **c** The inhibition of HH signaling by Fab^6H3^ is dose dependent. The dose-dependent inhibition of HH signaling by Fab^6H3^ was measured using the Dual-Luciferase Reporter Assay in SHH-Light II cells. Data are represented as mean ± SD (*n* = 3). Data analysis was performed using GraphPad Prism 10. **d** Overall structure of PTCH1*–Fab^6H3^ complex. The heavy chain, light chain of Fab^6H3^, ECD1, ECD2 and transmembrane domain of PTCH1* are colored in blue, light blue, cyan, yellow and green, respectively. **e** Interaction details between ECD1 of PTCH1* and Fab^6H3^. The hydrophilic interactions are shown by dashed lines. **f** Superposition of PTCH1*-Fab^6H3^ complex with each PTCH1* molecule of the 2:1 PTCH1*–SHH-N complex(PDB: 6E1H). **g** Superposition of PTCH1–Tl23 complex (PDB: 7K65) with PTCH1*–Fab^6H3^ complex (left) and apo PTCH1 (right, PDB: 6MG8). The conformational changes of PTCH1-ECD1-α5 and rotation of PTCH1-ECD2 triggered by Tl23 compared to apo PTCH1 are indicated. **h** Fab^6H3^, but not Ab^6H3^, can inhibit PTCH1 endocytosis. PTCH1-Flag transiently-transfected HEK293T cells were treated with PBS, native SHH-N, Ab^6H3^, and Fab^6H3^, respectively, for 3 h. After treatment, cell-surface PTCH1-Flag was labeled with Sulfo-NHS-SS-biotin and pulled down using streptavidin-agarose resin. Total and surface (non-internalized) PTCH1 were detected using anti-FLAG antibody. Endogenous β-actin was used as an internal control. **i**, **j** Relative *Gli1* mRNA level of kidney (**i**) and skin (**j**) from the Fab^6H3^ treatment group compared to PBS treatment group. Fab^6H3^ was generated with the Ab^6H3^ purified from hybridoma cells. Each point was an average of 4 technical replicates of RT-PCR. Data are represented as mean ± SD (*n* = 6). Two-sided *t*-test was performed between the PBS treatment group and Fab^6H3^ treatment group by GraphPad Prism 10. *P* values are labeled.

Our sequencing results showed that the variable regions of these three mAbs are very similar (Supplementary Fig. S3); Fab^6H3^ was purified with the highest yield among the three Fabs, and we used it as a representative of these Fabs in further experiments. The IC_50_ of Fab^6H3^ to PTCH1 is about 77.8 nM measured by the HH signaling assay (Fig. 1c). To uncover the interaction details between PTCH1 and Fab^6H3^, we assembled the complex of human PTCH1* and Fab^6H3^. The purified complex was well-behaved on gel filtration and was suitable for the structural investigation by cryogenic electron microscopy (cryo-EM) (Supplementary Fig. S4a). The structure of this complex was determined at 3.7-Å resolution (Fig. 1d; Supplementary Fig. S4b–f and Table S1). The structure reveals that Fab^6H3^ binds to the ECD1 of PTCH1 (Fig. 1d). The interface area between Fab^6H3^ and PTCH1 is ~900 Å^2^. Residues Asp50, Tyr51, Asp73, Asp122 of the heavy chain form the hydrophilic interactions with residues Tyr379, Glu380, Arg255 and Arg195 of the PTCH1-ECD1 respectively, while residue His54 of the light chain has hydrophilic contacts with Try191 and Asn194 of PTCH1 (Fig. 1e).

The conformation of PTCH1 in the PTCH1-Fab^6H3^ complex is similar to that of the two PTCH1 molecules (RMSD = ~1.0–1.2 Å) in the PTCH1*-SHH 2:1 complex (Supplementary Fig. S5a, PDB:6E1H). Interestingly, structural comparison shows that Fab^6H3^ interferes with both the palmitate-dominated interface between SHH and PTCH1a, and the Ca^2+^-mediated interface between SHH and PTCH1b (Fig. 1f). Structural comparison of the PTCH1-Fab^6H3^ complex with structure of Tl23-bound PTCH1 shows that Tl23 binds to ECD1-α5 and α6 (Fig. 1g); in contrast, Fab^6H3^ does not bind to these two structural elements (Fig. 1e). A previous study shows that Tl23 triggers conformational changes in ECD1-α5 and a rotation of ECD2 around its connection to the TM region by 5° toward ECD1 compared to that of apo murine PTCH1 (Fig. 1g). These shifts cause a narrowing of the intramolecular sterol conduit and subsequent abolishment of the PTCH1 transport activity^14^. However, Fab^6H3^ neither binds to ECD1-α5 nor leads to a rotation of ECD2 (Fig. 1f), suggesting that Fab^6H3^, unlike Tl23, may not affect the potential cholesterol transport activity of PTCH1. Moreover, our endocytosis experiment showed that Fab^6H3^ retains more PTCH1 on the cell surface compared to other reagents (Fig. 1h).

In the TMs of murine PTCH1, there is a charged triad consisting of D499, D500, and E1081, which contributes to the transport activity of PTCH1^4,10^. In the Tl23-bound state, a salt bridge forms between D499 and H1085; in contrast, this salt bridge is not observed in the apo state of human PTCH1 (Supplementary Fig. S5b). In our PTCH1-Fab^6H3^ structure, owing to resolution limitations or the inherent dynamics of the TM domain, the side chains of D513 and D514 (corresponding to murine D499 and D500) are not clearly visible (Supplementary Fig. S5c). As a result, we were unable to compare the formation of the salt bridge (D499-H1085) and the conformational changes of the charged triad in the TM domain upon Fab^6H3^ binding.

It is possible that Fab^6H3^ may be accessible to cells in mammalian tissues and function as an HH signal antagonist. Thus, the activity of Fab^6H3^ was determined through intraperitoneal (IP) injection in mice at a dose of 20 mg Fab^6H3^/kg. After 14 days, we sacrificed the mice and measured the mRNA levels of glioma-associated oncogene 1 (*Gli1*, essential for tumor growth and progression) from different organs using qPCR. Our results showed that the mRNA levels of Gli1 were significantly lower in kidneys of Fab^6H3^-treated mice compared to that in the control group (Fig. 1i), demonstrating the potential of Fab^6H3^ as an HH signaling inhibitor in vivo. We did not detect a consistent reduction of Gli1 mRNA levels in the skin from two individual experiments (Fig. 1j; Supplementary Fig. S6). The reason why Fab^6H3^ has a greater effect on HH signaling in the kidneys and whether it can prevent the growth of the kidney tumors require further investigations.

Current therapeutics that target the HH signaling pathway primarily consist of small molecule antagonists of SMO^2^. These molecules have been widely used for treating cancers driven by excessive HH pathway activity, specifically through targeting primary tumor cells. The side effects of these molecules include muscle spasms, hair loss, decreased appetite, nausea, or vomiting. Here, we developed a series of conformation-selective anti-PTCH1 reagents capable of binding to PTCH1 and subsequently repressing the HH signaling in cells and mouse kidneys. They may target HH signaling more specifically through the direct interaction with PTCH1, thus avoiding off-target side effects. However, the half-life of Fab is much shorter than that of Ab, and the essential modifications on Fab may be applied to extend the half-life of Fab in vivo.

A previous study identified the Tl23 nanobody, which acts as a partial agonist of the HH pathway^14^. Tl23 disrupts the cholesterol transport channel of PTCH1, leading to the accumulation of cholesterol in cilia to activate the HH signal. In comparison to Tl23, Fab^6H3^ binds to a different region of the ECD1 of PTCH1 and does not induce a conformational change in PTCH1 (Fig. 1e, g). It is possible that one antibody can bind to two PTCH1 molecules, which is akin to HH-PTCH1 signaling-competent complex to trigger the endocytosis of the PTCH1, activating the signaling^9^. However, our data showed that Fab^6H3^ prevents the HH binding and retains the PTCH1 on the cell surface presumably to keep the low cholesterol concentration in the membrane, thus inhibiting the signaling. The different mechanisms between Tl23 and Fab^6H3^ result in opposite functions for these two reagents, suggesting that targeting different regions of ECD1 with distinct reagents can modulate HH signaling.

## Supplementary information

Supplementary Information
