# Supplementary material for: Inhibiting hedgehog signal by a patched-1 antibody
Source: Cell Discov. 2025 Mar 25;11:29. doi: 10.1038/s41421-025-00772-6 (PMC11933295; doi:10.1038/s41421-025-00772-6)
Supplement: Supplementary file 1 — Supplementary Information [file 41421_2025_772_MOESM1_ESM.pdf]

Supplementary Information for

Inhibiting Hedgehog Signal by a Patched-1 Antibody

Qinli Hu, Xiaofeng Qi, * Linda Donnelly, and Xiaochun Li*

*Correspondence: xiaofeng.qi@utsouthwestern.edu (X.Q.) or xiaochun.li@utsouthwestern.edu (X.L.)

Materials and Methods

Antibody development

The human PTCH1* protein used for mouse immunization was purified and reconstituted with amphipols A8-35 in phosphate-buffered saline (PBS), as previously described [1]. Male New Zealand Black NZBWF1/J mice (Jackson Laboratory, strain no. 100008) aged 6–8 weeks were immunized with human PTCH1*. All research protocols involving mice were reviewed and approved by the Institutional Animal Care and Use Committee (IACUC) at the University of Texas Southwestern Medical Center. Following one primary injection and six booster injections, the splenic B lymphocytes from the immunized mouse were fused with SP2-mIL6 mouse cells (ATCC, CRL-2016) to generate hybridoma cells. To screen for conformation-specific PTCH1 antibodies, we performed enzyme-linked immunosorbent assays (ELISA) using native PTCH1* and western blotting with denatured PTCH1*. This process yielded three monoclonal antibodies (mAbs), which we named Ab^{6H3}, Ab^{4G8}, and Ab^{4H2}. For mAb cloning, total RNA was isolated from the hybridoma using an RNA extraction kit (Qiagen) and subjected to reverse transcription using the Superscript III reverse transcription kit (Invitrogen). The resulting cDNA was diluted 20-fold and used as a template for PCR with degenerate primers to amplify the variable regions of the mAbs. The sequencing results of the PCR products were analyzed using the IMGT database (http://www.imgt.org/) to identify the variable regions of the light and heavy chains.

Antibody and Fab expression and purification from hybridoma cells

The medium used to culture the hybridoma cells was harvested and passed through protein G resin (Cytiva) twice to capture the Abs. After washing the resin with Buffer A (20 mM HEPES, 150 mM NaCl, pH 7.5), the Abs were eluted with 100 mM glycine (pH 3.0). The eluate was immediately supplemented with 100 mM HEPES (pH 7.5), followed by overnight dialysis against Buffer A. To generate the Fab fragments, the purified Abs were concentrated and digested with papain protease at a papain-to-antibody ratio of 1:50 (w/w) in Buffer B (20 mM HEPES, 150 mM NaCl, 10 mM Cys-HCl, 10 mM β-mercaptoethanol, 10 mM EDTA, pH 7.5) at 37°C for 2 hours. The reaction was stopped by adding 20 mM iodoacetamide at room temperature (RT) for 10 minutes. The mixture was then purified using a HiTrap Q column (Cytiva). The peak fractions containing Fab

fragments were collected, dialyzed against PBS, concentrated to ~5 mg/ml, and frozen at -80°C for future use.

Fab^{6H3} expression and purification from *E. coli*

To express Fab^{6H3} in *E. coli*, the codon-optimized variable regions of Ab^{6H3} were cloned into an anti-BRIL Fab bacterial expression construct [2], replacing the existing variable regions of the anti-BRIL Fab. The resulting Fab^{6H3} construct includes an N-terminal signal peptide in both the light and heavy chains and a C-terminal 6xHis tag in the heavy chain. *E. coli* BL21 (DE3) cells were transformed with the Fab^{6H3} construct and grown in LB medium at 37°C until the OD600 reached ~0.8. Expression was then induced with 0.5 mM IPTG overnight at 20°C. Fab^{6H3} was purified from the periplasmic space following a previous protocol [3]. Briefly, the bacterial pellet was resuspended in Buffer C (500 mM sucrose, 0.5 mM EDTA, 200 mM Tris-HCl, pH 8.0) at a ratio of ~25 ml of buffer per liter of bacteria. After stirring at RT for 30 minutes, two volumes of water were added, and the mixture was stirred for an additional 45 minutes. $MgCl_2$ was then added to a final concentration of 2 mM, followed by benzonase (Sigma) at a 1:100,000 (v/v) ratio. After 5 minutes, NaCl was added to a concentration of 150 mM, and imidazole was added to 20 mM. The mixture was centrifuged at 20,000 g for 15 minutes, and the resulting supernatant was passed through the Ni-NTA resin (Qiagen). The resin was washed with Buffer A supplemented with 20 mM imidazole, and Fab^{6H3} was eluted using Buffer A supplemented with 250 mM imidazole. The eluted protein was concentrated and further purified using a Superdex 200 Increase column (Cytiva) in PBS for subsequent experiments.

Pull down assay

Purified Flag-tagged PTCH1* protein [1] was conjugated to anti-Flag M2 resin (Sigma) and further incubated with purified His-tagged SHH-N [1] at 4°C for 1 hour in Buffer D (20 mM HEPES, 150 mM NaCl, 0.02% n-dodecyl-ß-D-maltoside (DDM), pH 7.5) supplemented with 1 mM $CaCl_2$. Full-length Ab^{4G8}, Ab^{4H2}, or Ab^{6H3} derived from hybridoma was then added to the mixture and incubated at 4°C for another hour. The resin was then spun down and washed three times with

Buffer D. The protein samples were eluted with Buffer D containing 0.1 mg/mL 3xFlag peptide and then loaded onto an SDS-PAGE gel for protein detection.

Cell based Fab^{6H3} titration assay

SHH-N conditioned medium was prepared as previously described [1]. SHH-Light II cells were used to assess the concentration-response inhibitory effects of Fab^{6H3}. The cells were serum-starved in DMEM with 0.5% FBS for 24 hours, then incubated with hybridoma-derived Fab^{6H3} at varying concentrations in combination with the HH-conditioned medium. After 24 hours, firefly and *Renilla* luciferase activities were measured using the Dual-Luciferase Reporter Assay System (Promega). Data analysis was performed using Prism 10 (GraphPad). Experiments were repeated at least three times with similar results.

Cell based Internalized assay

Human PTCH1 were cloned to pcDNA3.1 vector with a C-terminal Flag tag. The 293T cells were setup in 6-well plate pre-coated with by Poly-D-Lysine (Thermo Scientific). The PTCH1 plasmid (2μg/well) was then transfected using FuGENE HD Transfection Reagent (Promega). 24 hours post-transfection, the transfected cells were serum-starved in DMEM with 0.5% FBS. Then, the cells were treated with PBS, native SHH-N (R&D Systems) (1.8μg/ml), Ab^{6H3}(60 μg/ml), and Fab^{6H3} (60 μg/ml), respectively at 37°C for 3 hours. Sulfo-NHS-SS-biotin (Apexbio Technology LLC) was dissolved in CM-PBS (100 mg/L $MgCl_2 \cdot 6H_2O$ and 100 mg/L $CaCl_2$ in 1X PBS, pH7.4) and incubated with the transfected cells on ice for 30 minutes. The labeling reaction was stopped by incubating the cells with quenching buffer (20 mM glycine in CM-PBS) on ice for 30 minutes. The cells were resuspended with quenching buffer (1 mL per well). 100 μL RIPA buffer (Thermo Scientific) was added to 100 μL cells as the input sample. The remaining cells were incubated with 1% DDM (n-Dodecyl-β-D-maltoside) (Anatrace) at 4 °C for 30 minutes to lyse the cells. The cell pellet was removed by centrifuged at 14,000 x g for 10 minutes. The supernatant was incubated with 40 μL streptavidin-agarose resin (Thermo Scientific) at 4°C for 30 minutes. Resin was washed three times with wash buffer (20 mM Tris,150 mM NaCl, 0.02% DDM, pH8.0) and then incubated with 60 μL 2X loading buffer, containing 8 M Urea, 10% SDS, and 40 mM DTT for western blot

analysis. Total PTCH1 and surface (non-internalized) PTCH1 were detected by anti-FLAG tag antibody (MBL International), and endogenous Beta-actin was used as an internal control, detected with an anti-beta-actin antibody (Santa Cruz Biotechnology). Each experiment was performed at least three times with similar results.

Cryo-EM sample preparation and data acquisition

Hybridoma-derived Fab^{6H3} was mixed with purified PTCH1* in Buffer D at a molar ratio of 1:1.5 and incubated for 1 hour. The mixture was then injected onto a Superdex 200 Increase column (Cytiva) and eluted in Buffer E (20 mM HEPES, 150 mM NaCl, 0.06% digitonin, pH 7.5). Fractions containing the PTCH1*–Fab^{6H3} complex were collected and concentrated to ~ 5 mg/ml for cryo-EM studies. The protein sample was applied to Quantifoil R1.2/1.3 400-mesh Au holey carbon grids (Quantifoil), blotted using a Vitrobot Mark IV (FEI), and flash-frozen in liquid ethane. Data were collected using a 300 kV Titan Krios (FEI) equipped with a Gatan K3 Summit direct electron detector (Gatan) and an energy filter (slit width 20 eV). Raw movie stacks were acquired at a pixel size of 0.833 Å and a nominal defocus range of 1–2 µm, with a total dose of ~60 electrons per $Å^2$.

Cryo-EM Data Processing and structure refinement

The movie stacks were motion-corrected using MotionCor2 [4] and the contrast transfer function (CTF) was estimated with CTFFIND4 [5]. Auto-picking of particles was performed using Relion3 [6], after which low-quality images and false-positive particles were manually removed. The remaining particles were extracted and processed through 2D classification, 3D classification, 3D refinement, and Bayesian polishing in Relion3. The polished particles were then imported into cryoSPARC [7] for further 2D classification. Particles from 2D classes with a clean background were selected for ab initio model generation, followed by heterogeneous refinement in cryoSPARC. The best class from the heterogeneous refinement was subjected to non-uniform refinement and final local refinement in cryoSPARC.

A previously determined PTCH1* structure (PDB: 6E1H) and a SWISS-MODEL [8] predicted structure of Fab^{6H3} were docked into the cryo-EM map as the initial models. The structural model was manually built and adjusted in COOT [9], followed by real-space refinement using PHENIX [10]. The final model was validated using PHENIX. Structural figures were generated using PyMOL (http://www.pymol.org) and ChimeraX.

Cell based HH signaling inhibition assay

SHH-N conditioned medium was prepared as previously described [1]. SHH-Light II cells were used to assess the inhibitory effects of the Fabs. The cells were incubated with hybridoma-derived Fab^{4G8}, Fab^{4H2}, Fab^{6H3}, or a control Fab (Fab^{NPC1}) at a final concentration of 60 μg/mL for 6 hours before being treated with the conditioned medium. After 24 hours, firefly and *Renilla* luciferase activities were measured using the Dual-Luciferase Reporter Assay System (Promega). Data analysis was performed using Prism 10 (GraphPad). Results are presented as mean ± S.D., with experiments repeated at least three times with similar results.

In vivo HH signaling inhibition assay

Female C57BL/6J mice (Jackson Laboratory), aged 7-8 weeks, were injected intraperitoneally (IP) with 400 μg (150 μL) of Fab^{6H3}, obtained from either hybridoma or bacterial sources as described above. The control group received an equivalent volume of PBS. In the bacteria-derived Fab^{6H3} group, intraperitoneal injections were administered every other day, while in the hybridoma-derived Fab^{6H3} group, injections were given daily, both for a duration of two weeks. After the treatment period, various organs were collected for RNA extraction and reverse transcription. GLI1 mRNA levels were measured by reverse-transcription quantitative PCR (RT-qPCR), using mouse GAPDH as an internal control as described before [11]. The primers used were as follows:

GLI1 forward, 5'-CAATTTCAACCCCTCCTCCTCT;

GLI1 reverse, 5'-AGGTGCAAAGCCAGATCCATA;

GAPDH forward, 5'-CATTTGCAGTGGCAAAGTGGAG;

GAPDH reverse, 5'-ACCCCATTTGATGTTAGTGGGG.

RT-qPCR was performed using either a QuantStudio 5 Real-Time PCR System (Thermo Fisher Scientific) or a ViiA 7 Real-Time PCR System (Applied Biosystems) with SYBR Green (Bio-Rad). Relative GLI1 transcript levels were calculated using the ΔΔCt method.

References:

1 Qi, X., Schmiege, P., Coutavas, E., Wang, J. & Li, X. Structures of human Patched and its complex with native palmitoylated sonic hedgehog. *Nature* **560**, 128-132 (2018).

2 Mukherjee, S. *et al.* Synthetic antibodies against BRIL as universal fiducial marks for single-particle cryoEM structure determination of membrane proteins. *Nature communications* **11**, 1598 (2020).

3 Zhang, Y. *et al.* Hedgehog pathway activation through nanobody-mediated conformational blockade of the Patched sterol conduit. *Proceedings of the National Academy of Sciences of the United States of America* **117**(46):28838-28846 (2020).

4 Zheng, S. Q. *et al.* MotionCor2: anisotropic correction of beam-induced motion for improved cryo-electron microscopy. *Nature methods* **14**, 331-332 (2017).

5 Rohou, A. & Grigorieff, N. CTFFIND4: Fast and accurate defocus estimation from electron micrographs. *J. Struct. Biol.* **192**, 216-221 (2015).

6 Zivanov, J. *et al.* New tools for automated high-resolution cryo-EM structure determination in RELION-3. *eLife* **7** :e42166 (2018).

7 Punjani, A., Rubinstein, J. L., Fleet, D. J. & Brubaker, M. A. cryoSPARC: algorithms for rapid unsupervised cryo-EM structure determination. *Nat. Methods* **14**, 290-296 (2017).

8 Waterhouse, A. *et al.* SWISS-MODEL: homology modelling of protein structures and complexes. *Nucleic acids research* **46**, W296-W303 (2018).

9 Emsley, P. & Cowtan, K. Coot: model-building tools for molecular graphics. *Acta crystallographica. Section D, Biological crystallography* **60**, 2126-2132 (2004).

10 Adams, P. D. *et al.* PHENIX: a comprehensive Python-based system for macromolecular structure solution. *Acta crystallographica. Section D, Biological crystallography* **66**, 213-221 (2010).

11 Qi, X., Friedberg, L., De Bose-Boyd, R., Long, T. & Li, X. Sterols in an intramolecular channel of Smoothened mediate Hedgehog signaling. *Nature chemical biology* **16**, 1368-1375 (2020).

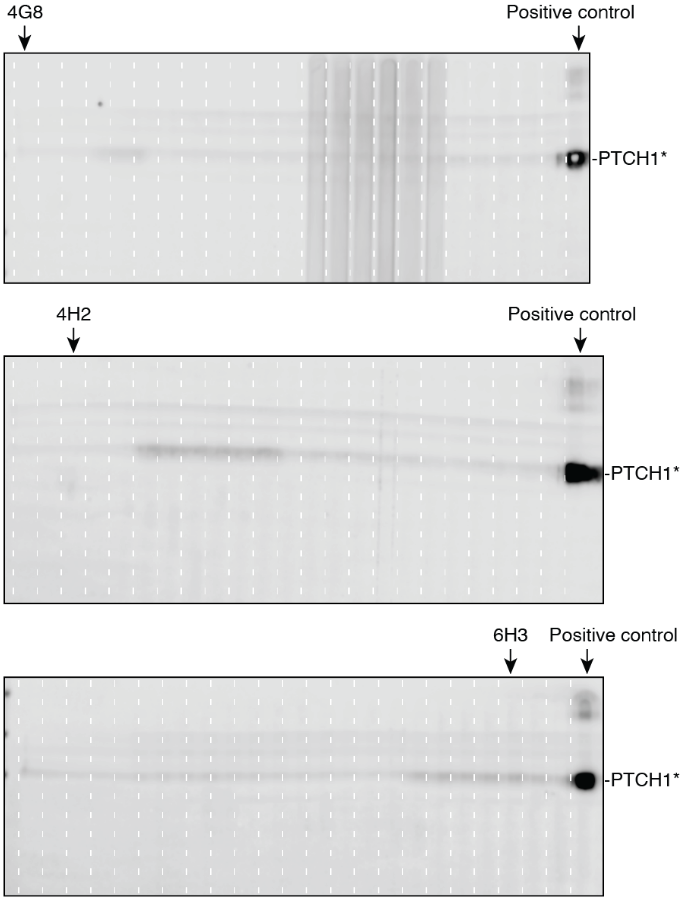

Supplementary Fig. S1 Western blotting screening of the PTCH1 antibodies.

Denatured Flag-tagged PTCH1* was loaded into each lane and incubated with medium from different hybridoma subclones. A commercial anti-Flag antibody (MBL International) was used as a positive control. The three antibodies characterized in this work, 4G8, 4H2 and 6H3, are indicated.

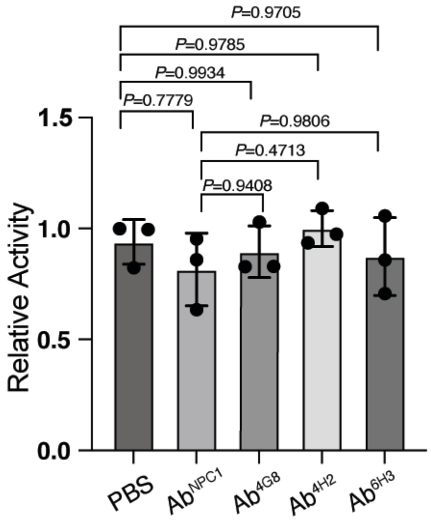

Supplementary Fig. S2 Abs cannot inhibit the HH signal validated by HH signaling assay.

The HH signal was measured using Dual-Luciferase Reporter Assay in SHH-Light II cells. PBS buffer and an anti-NPC1 Antibody (Ab^{NPC1}) are used as negative controls. Data are represented as mean ± S.D. (n=3). One-way ANOVA was performed between the control groups and each of the experimental group by GraphPad Prism 10. *P* values are labeled.

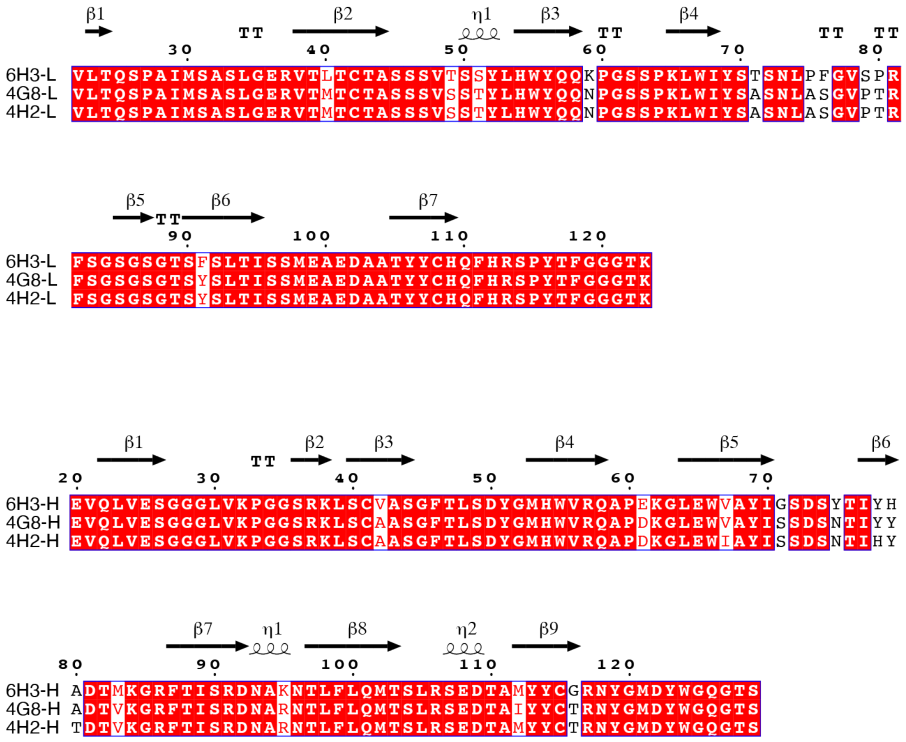

Supplementary Fig. S3 Sequence alignment of the variable regions of Fab^{6H3}, Fab^{4G8} and Fab^{4H2}.

Sequence alignment of the variable regions of the light chains (L, up) and heavy chains (H, down) of the three Fabs. The residue number and secondary structure are on the top of sequences. The conserved residues are highlighted in red background.

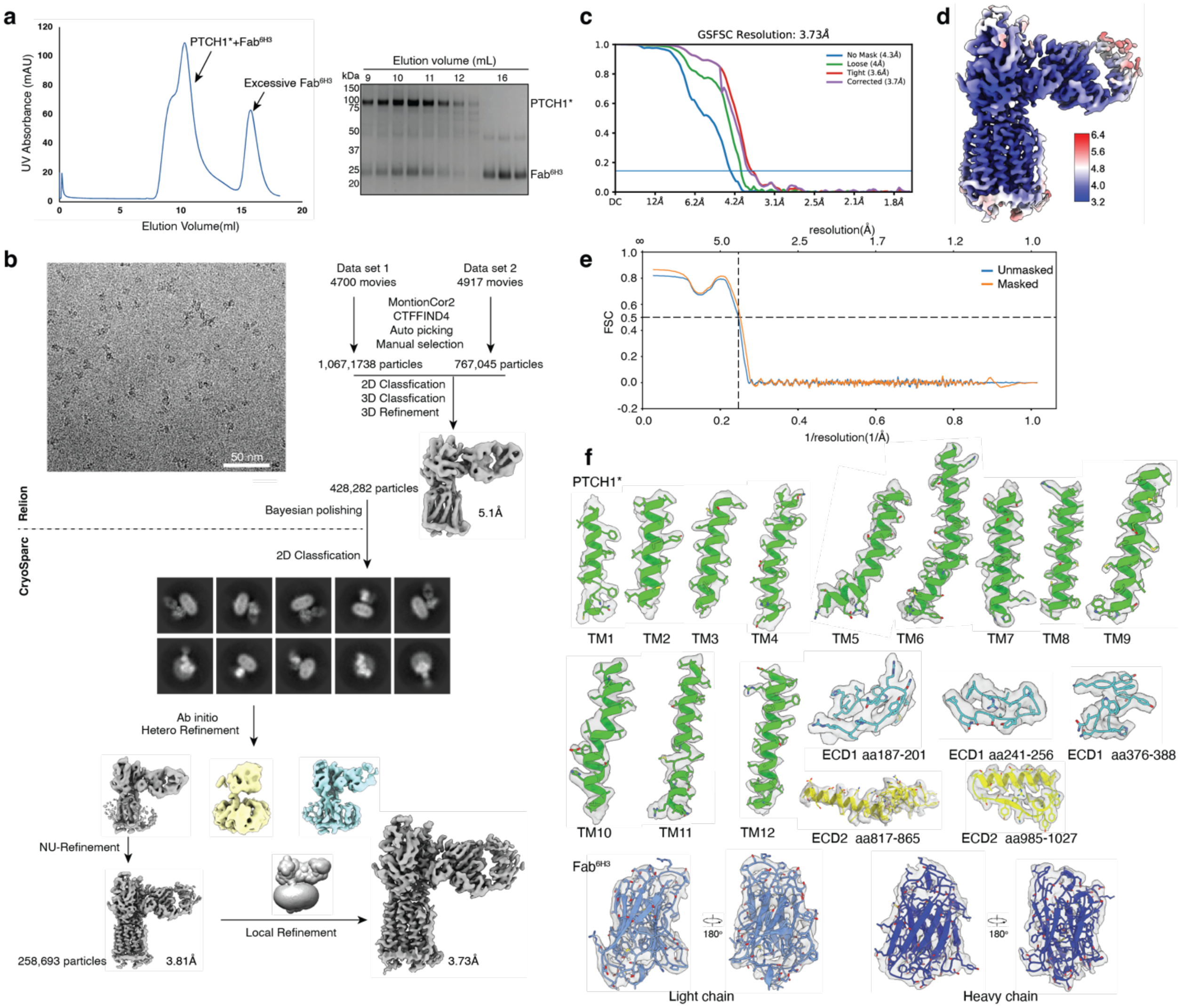

Supplementary Fig. S4 Biochemical and cryo-EM analyses of PTCH1*-Fab^{6H3} complex

a) Representative gel-filtration chromatogram and SDS-PAGE of PTCH1*-Fab^{6H3} complex.

b) Flow chart of cryo-EM data processing procedures of PTCH1*-Fab^{6H3} complex.

c) Fourier shell correlation (FSC) curves between two half maps of PTCH1*-Fab^{6H3} complex.

d) Local resolution of cryo-EM map of PTCH1*-Fab^{6H3} complex. Maps are colored according to local resolution, estimated by cryoSPARC.

e) The map-model Fourier Shell Correlation (FSC) curve of PTCH1*-Fab^{6H3} complex.

f) Cryo-EM map of the major structural elements of PTCH1*-Fab^{6H3} complex.

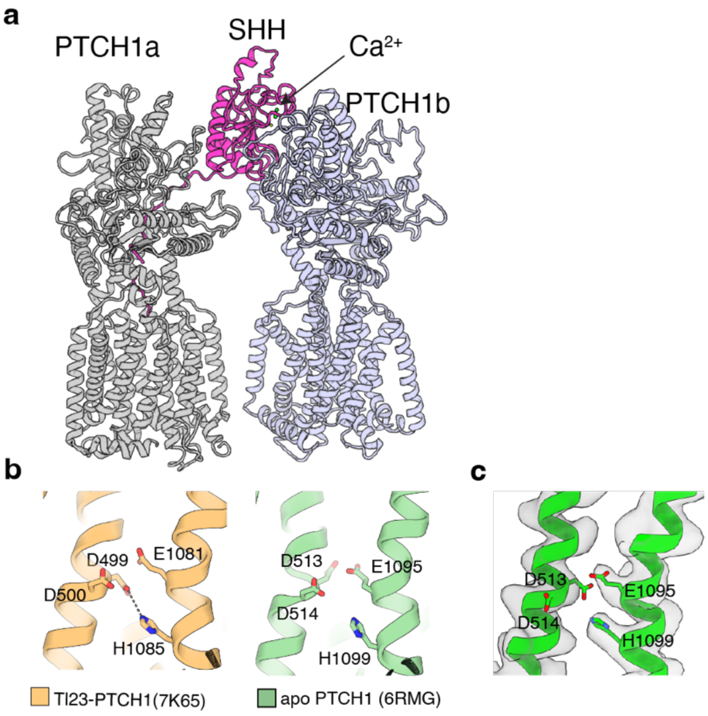

Supplementary Fig. S5 Structure of PTCH1*–SHH-N complex and side chain map density of D513 and D514.

a) Overall structure of 2:1 PTCH1*–SHH-N complex (PDB: 6E1H).

b) Side chain interaction comparison between Tl23-PTCH1 complex and apo PTCH1. The hydrophilic interactions are indicated by a dashed line.

c) Side chain map density of D513, D514, E1095 and H1099 in PTCH1*-Fab^{6H3} complex.

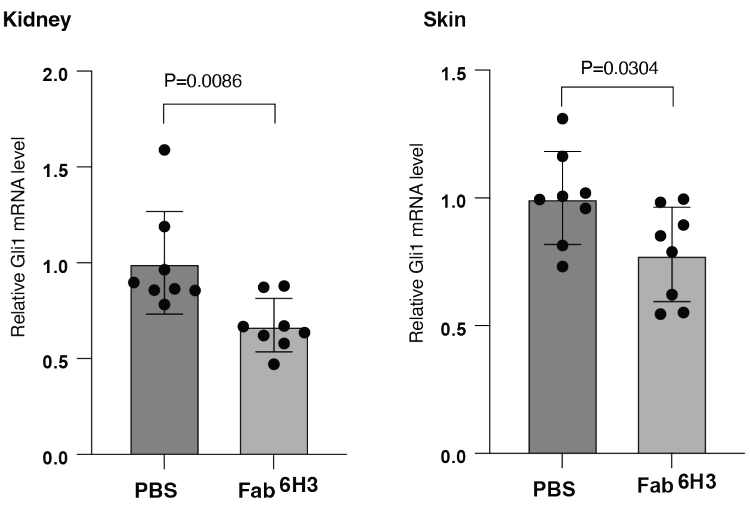

Supplementary Fig. S6 Biological repeats of relative Gli1 mRNA levels of the kidneys and skin from mice treated with the Fab^{6H3}.

Relative Gli1 mRNA levels of kidneys and skin from the bacteria-derived Fab^{6H3} treatment group compared to the PBS treatment group. Fab^{6H3} was purified from *E. coli*. Each point was an average of 4 technical replicates of RT-PCR. Data are represented as mean ± S.D. (n = 8). Two-sided t test was performed between the PBS treatment group and Fab^{6H3} treatment group by GraphPad Prism 10. *P* values are labeled.

Supplementary Table S1: Cryo-EM data collection, refinement and validation statistics

	PTCH1-Fab^{6H3} (EMDB-48568) (PDB-9MS8)
**Data collection and processing**	
Nominal Magnification	105 k
Voltage (kV)	300
Electron exposure (e–/Å^2)	60
Defocus range (μm)	-1.0 to -2.0
Pixel size (Å)	0.833
Symmetry imposed	C1
Initial particle images (no.)	1,834,783
Final particle images (no.)	258,693
Map resolution (Å)	3.73
FSC threshold	0.143
**Refinement**	
Initial model used (PDB code)	6E1H
Model resolution (Å)	4.0
FSC threshold	0.5
Map sharpening *B* factor (Å^2)	-146.2
Model composition	
Non-hydrogen atoms	9292
Protein residues	1189
Ligands	0
B factors (Å^2)	
Protein	97.88
Ligand	-
R.m.s. deviations	
Bond lengths (Å)	0.003
Bond angles (°)	0.563
Validation	
MolProbity score	2.15
Clashscore	7.92
Poor rotamers (%)	1.72
Ramachandran plot	
Favored (%)	90.50
Allowed (%)	9.50
Disallowed (%)	0
